# Designing the ideal model for assessment of wound contamination after gunshot injuries: a comparative experimental study

**DOI:** 10.1186/1471-2482-12-6

**Published:** 2012-04-10

**Authors:** Constantin von See, Majeed Rana, Marcus Stoetzer, Horst Kokemueller, Martin Ruecker, Nils-Claudius Gellrich

**Affiliations:** 1 Department of Oral and Maxillofacial Surgery, Hannover Medical School, Hannover, Germany

**Keywords:** Forensic science, Wound infection, Gunshot, Projectile, Gelatin

## Abstract

**Background:**

Modern high-velocity projectiles produce temporary cavities and can thus cause extensive tissue destruction along the bullet path. It is still unclear whether gelatin blocks, which are used as a well-accepted tissue simulant, allow the effects of projectiles to be adequately investigated and how these effects are influenced by caliber size.

**Method:**

Barium titanate particles were distributed throughout a test chamber for an assessment of wound contamination. We fired .22-caliber Magnum bullets first into gelatin blocks and then into porcine hind limbs placed behind the chamber. Two other types of bullets (.222-caliber bullets and 6.5 × 57 mm cartridges) were then shot into porcine hind limbs. Permanent and temporary wound cavities as well as the spatial distribution of barium titanate particles in relation to the bullet path were evaluated radiologically.

**Results:**

A comparison of the gelatin blocks and hind limbs showed significant differences (*p *< 0.05) in the mean results for all parameters. There were significant differences between the bullets of different calibers in the depth to which barium titanate particles penetrated the porcine hind limbs. Almost no particles, however, were found at a penetration depth of 10 cm or more. By contrast, gas cavities were detected along the entire bullet path.

**Conclusion:**

Gelatin is only of limited value for evaluating the path of high-velocity projectiles and the contamination of wounds by exogenous particles. There is a direct relationship between the presence of gas cavities in the tissue along the bullet path and caliber size. These cavities, however, are only mildly contaminated by exogenous particles.

## Background

Gunshot injuries not only cause direct tissue damage but often also lead to contamination by foreign material along the bullets path [1,2]. The role of exogenous particles that contaminate a wound cavity and the role of non-bacterial tissue damage resulting from sterile necrosis remain controversial in the literature [3]. As a result, the ballistic effects of gunshot injuries are a matter of controversy as well [4,5]. There is, however, general agreement that high-velocity projectiles cause cavitation effects and tissue destruction and thus produce areas of tissue necrosis with different diameters radially from the bullet path [6]. In gunshot injuries, the extent of cavitation effects generally depends on projectile velocity and caliber [7,8]. Different calibers mainly differ in projectile diameter and weight. These factors play a key role in the transfer of energy to the target [9,10]. When a bullet enters a body, energy is imparted to the tissue via a radial pressure wave and cavitation effects [11,12]. These physical effects lead to the formation of a temporary wound cavity and the creation of negative pressure. The extent to which this suction effect draws exogenous particles into the temporary wound cavity is yet unclear.

Clinical radiological examinations of gunshot injuries often show the presence of gas cavities in the tissue along the bullet path which provide first information about the temporary wound cavity. Radiological and clinical examinations alone, however, have thus far not been able to reliably demonstrate the presence or absence of exogenous particles in these gas cavities. Whether or not a wound is contaminated, however, might help to estimate the caliber and ammunition of the used rifle in gunshot wounds.

It is difficult to standardize the effects of gunshot injuries on tissue not least because it is virtually impossible to perform direct systematic examinations of gunshot injuries in living tissues. For this reason, there are many well-established models for studying gunshot injuries [13,14]. Ordnance gelatin is one of the best-investigated materials for examining the behavior of tissue when hit by a bullet [15]. Of all materials available, gelatin is believed to be the one that most closely simulates the elastic behavior of tissue. Although there are many studies addressing the formation of temporary wound cavities, the suitability of gelatin for investigating wound contamination has not yet been conclusively proven.

The objective of this study was therefore to compare bullet paths and wound contamination in gelatin blocks and tissue and to assess the influence of caliber size.

## Methods

The study was approved by the local ethics committee at the Hannover Medical School, Germany (EK/2009).

### Experimental set-up

The firing tests were performed with bullets of different calibers, i.e. .22-caliber Magnum bullets, .222-caliber Winchester bullets and 6.5 × 57 mm bullets (all RWS, Rottweil, Germany). All bullets were full metal jacketed. The projectiles had different weights, but comparable similar initial velocities (v_0 _945-970 m/s).

The experimental set-up used a firing apparatus with a test chamber and a rifle support. Before each shot, 5.0 g of barium titanate (Aldrich, Steinheim, Germany) with a particle size of 3-6 μm were placed inside the chamber. Three air pressure valves were attached to the chamber (at the bottom and on the left and right sides) and connected to an air compressor. The porcine hind limbs and the gelatin blocks were mounted on a support frame that was positioned directly behind the chamber in such a way that the gelatin blocks and hind limbs could be placed in the line of fire.

### Specimens

The gelatin blocks that were used in our experiments consisted of 20% porcine gelatin (Merck, Darmstadt, Germany) and water. The size of the blocks was 120 mm by 120 mm by 180 mm. The hind limbs were obtained from freshly slaughtered pigs with a total weight of approximately 65 kg. They were separated from the body in the median plane in order to facilitate their handling during the tests.

The experimental set-up described above was used to fire .22-caliber Magnum bullets into gelatin blocks (n = 6) and hind limbs (n = 6). The other two types of bullets (.222-caliber Winchester and 6.57 × 57) were fired only into porcine hind limbs (n = 6 each).

### Procedure

One shot was fired into each gelatin block and hind limb. At the time of firing, the gelatin blocks had a temperature ranging between +8°C and +10°C. They were mounted on the support frame in such a way that the bullet passed right through the block. Before each shot, 5.0 g of barium titanate were placed inside the test chamber. An air-permeable filter paper was inserted in the chamber to separate the interior of the chamber from the barrel of the rifle. A gelatin block was placed directly against the opposite side of the chamber. Shortly before a shot was fired, the compressor was used to expose the chamber to a blast of compressed air (0.5 bar) and to generate a cloud of barium titanate particles inside the chamber. A shot was then fired from the rifle that was mounted on the rifle support.

Barium titanate was again placed inside the chamber and the porcine hind limbs were positioned against the chamber in such a way that they tightly sealed the chamber. The chamber was again exposed to a blast of compressed air and a shot was fired from each of the three rifles into the hind limbs.

After each shot, 16-slice computed tomography (CT) scans (GE Medical Systems, Lightspeed, United States) of the gelatin blocks and hind limbs were obtained. All scans were performed at 120 kV and 200 mA.

### Data evaluation

The data obtained were made available in a digital format (DICOM) and transferred to a personal computer for further analysis. Voxim software (Voxim, IVS Solution, Germany) was used for statistical analysis. For this purpose, the gelatin blocks were virtually cut into 20-mm-thick vertical slices and evaluated. Once the center of the gelatin block was identified, the mean diameter of the permanent cavity was calculated. The distances from eight sites on the wall of the permanent cavity to the center of the block were measured radially and the results were averaged for each vertical slice.

Likewise, the distances from eight sites on the wall of the temporary cavity to the center of the block were measured radially and the results were averaged for each vertical slice. In order to determine the depth of infiltration in the gelatin blocks, the distances between barium titanate particles detected inside the temporary wound cavity and the center of the gelatin block at eight sites was measured. The results were averaged for each vertical slice.

Similarly, the permanent and temporary cavities and the distribution of barium titanate particles inside the temporary wound cavity were assessed in the hind limbs. For this purpose, the path of the bullet was determined precisely on the basis of radiological parameters such as the presence of gas and the entrance and exit wounds. Two-cm-thick CT slices were obtained perpendicular to the bullet path and analyzed using the aforementioned parameters.

### Statistical analysis

Results are expressed as means ± SEM. Differences between groups were evaluated with an analysis of variance (ANOVA) on ranks. Differences within groups were analyzed by one-way repeated measures ANOVA. Student-Newman-Keuls or Dunn's post-hoc tests were used to isolate specific differences. A p-value < 0.05 was considered significant.

## Results

After firing into the gelatin blocks and hind limbs, no projectiles or projectile fragments were detected in the specimens. Entrance and exit sites were found in all specimens.

### Comparative analysis of gelatin blocks and porcine hind limbs

**Figure 1:**
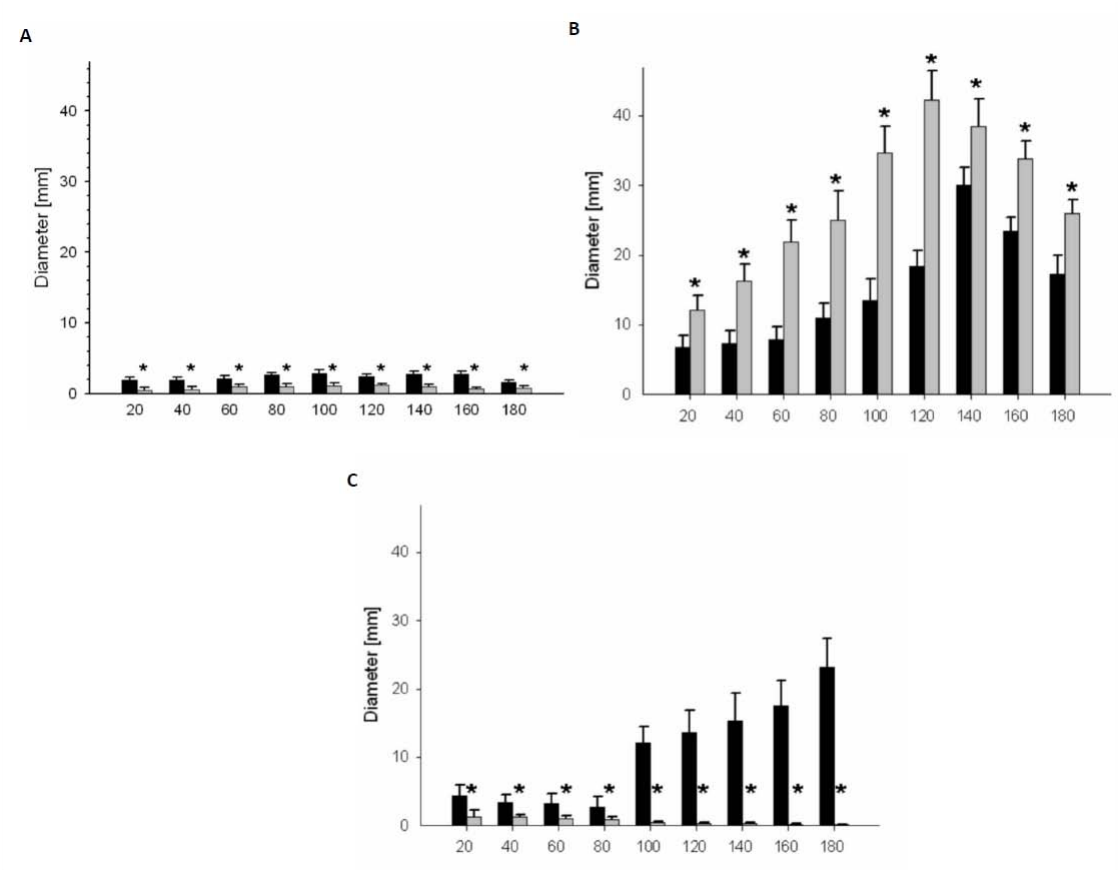
**Mean diameter for the permanent cavity **(A)**, the temporary cavity **(B) **and depth of infiltration of barium titanate particles along the bullet path **(C) **after firing with a .22 Magnum full metal jacketed projectile either in forensic gelatin blocks (black bar) or porcine hind limbs (grey bar)**. Comparison obtained for the gelatin blocks and porcine hind limbs revealed significant differences (*p *< 0.05) between the two groups of specimens in all three parameters investigated.

A comparison of the results obtained for the gelatin blocks and porcine hind limbs revealed significant differences (*p *< 0.05) between the two groups of specimens in all three parameters investigated. Along the entire bullet path, the mean diameter of the permanent wound cavity in the gelatin blocks was significantly larger than that measured in the hind legs (Figure 1A). The opposite was true for the temporary wound cavity. Along the entire path of the bullet, the mean diameter of the temporary cavity was larger in the hind limbs (Figure 1B). Barium titanate particles had penetrated more deeply along the bullet path in the gelatin blocks than in the hind limbs. It was interesting to note that the diameter of the area where barium titanate particles were detected increased from the entrance site to the exit site in the gelatin blocks. The opposite was true for the porcine hind limbs where the diameter of the area where barium titanate particles were detected decreased from the entrance site to the exit site (Figure 1C).

### Comparison of the different calibers in hind leg specimens

#### Permanent wound cavity along the bullet path

**Figure 2:**
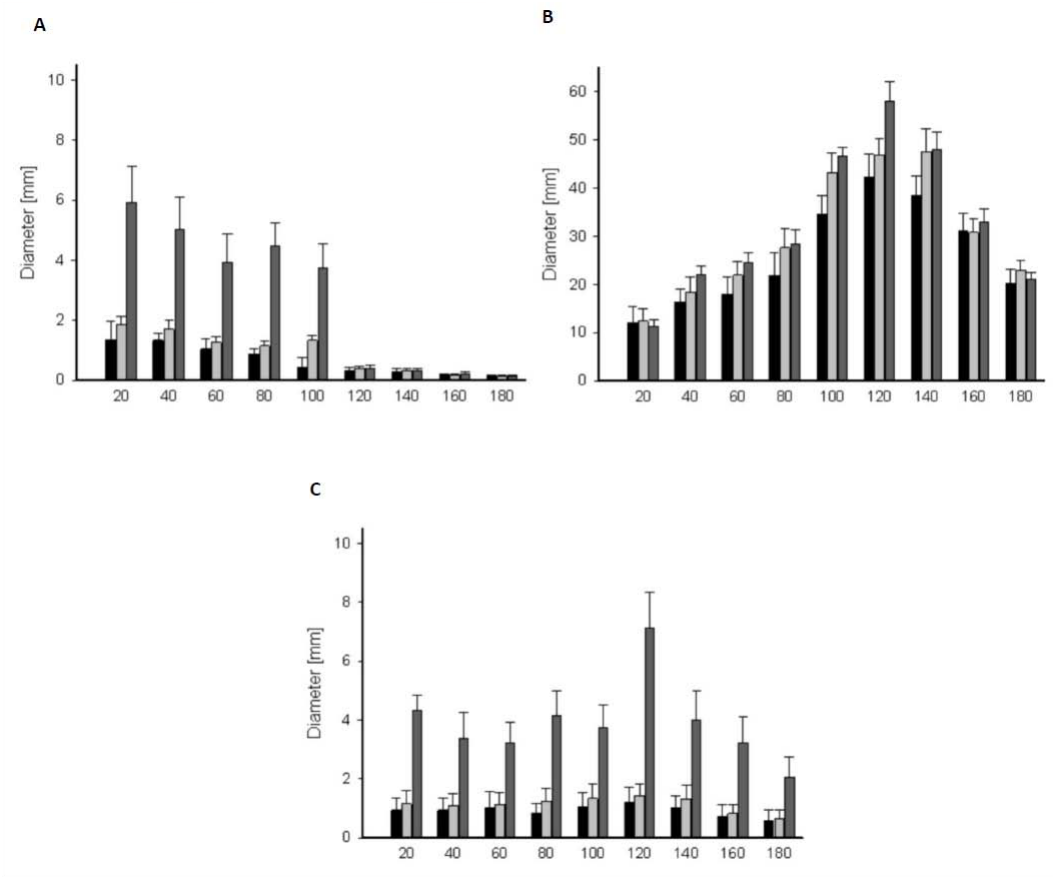
**Mean diameter of the permanent cavity **(A)**, the temporary cavity **(B) **and depth of infiltration of barium titanate particles along the bullet path **(C) **after firing at a porcine hind limb with either a .22 Magnum (black bar), .222-caliber Winchester (light grey bar) or 6.5 × 57 mm full metal jacketed bullets (dark grey bar)**.

There was a correlation between caliber size and the mean diameter of the permanent wound cavity along the bullet path. Permanent cavity diameter was found to increase with caliber. When the .22-caliber bullet was used, the diameter of the permanent wound cavity was up to seven times smaller than the diameter of the cavity that was produced by a 6.5 × 57 mm cartridge (Figure 2A).

#### Temporary wound cavity

The results that we obtained for the diameter of the temporary wound cavity along the bullet path were similar to those obtained for the permanent cavity. The diameter increased from the entrance site, reached its maximum at a penetration depth of 120-140 mm and then decreased again (Figure 2B). A comparison of the diameters of the temporary cavities that were created with bullets of different calibers showed that the diameters changed in a similar manner along the bullet path and were almost identical at a depth of 20 mm. The maximum diameters, however, were significantly different. Irrespective of the caliber used, the diameters of the temporary cavities were significantly larger than those of the permanent wound cavities (*p *< 0.05).

#### Depth of infiltration of barium titanate particles along the bullet path

Irrespective of the caliber used, the diameter of the area where barium titanate particles were found continuously decreased from the entrance site to the exit site. The diameter of these areas depended on the caliber used and increased with caliber size. At a penetration depth of 100 mm or more, almost no barium titanate particles in the hind limbs were detectable (2 C).

## Discussion

A direct comparison of ordnance gelatin blocks and porcine hind limbs showed significant differences in permanent cavities, temporary cavities and the infiltration depth of barium titanate particles.

In porcine hind limbs, the diameter of the bullet path and the depth of infiltration of barium titanate particles along the bullet path increased with caliber. At a penetration depth of 100 mm or more, however, we detected almost no more exogenous particles in the hind limbs.

Gunshot injuries can be examined using a wide variety of methods such as high-frequency video analysis, animal testing, and shots into human tissue simulants [16-18]. Tissue simulants have different physical properties such as elasticity, the ability to regain their original shape, and density. Nevertheless, many basic systematic studies are conducted on tissue simulants in order to reduce the various influencing factors of projectiles. These studies are usually homogenously structured and provide reproducible results. The different substances that are used in these studies, however, show large differences especially in elasticity. Rutty et al. reported that the properties of gelatin blocks such as those used in our study make them an ideal model for investigations of the temporary wound cavity and possible cavitation effects [19]. The use of multi-slice CT imaging for detecting the presence of total crack lengths has been proved to be a reliable method of investigations of terminal ballistics [20]. Furthermore barium titanate particles in the wound allowed us to reliably analyze the contamination of wounds by exogenous particles and to determine the spatial distribution of these particles in relation to the bullet path.

Our study revealed not only significant differences between gelatin blocks and porcine hind limbs in terms of the permanent wound cavity, the temporary wound cavity and the infiltration of exogenous particles but even opposite trends in the parameters investigated. This clearly shows that the results obtained by firing projectiles and especially high-velocity projectiles into gelatin blocks can be transferred only to a limited extent to complex anatomical structures made of different tissues. The porcine hind limbs that we used in our study consisted primarily of skin with subcutaneous fat and muscle tissue. According to Jussila et al., the transferability of the results to other types of tissue is very limited [21].

In our study, we used high-velocity projectiles of different calibers that do not fragment. Although the projectiles exhibited similar velocities, they caused significantly different degrees of tissue destruction and wound contamination in porcine hind limbs. The diameter of the permanent and temporary wound cavities were found to increase with caliber size. The permanent wound cavity is caused by the sound wave preceding the bullet and the displacement of tissue by the projectile. In soft tissues, however, the permanent wound cavity often collapses behind the projectile on account of the elastic behavior and displacement of tissue [22]. High-velocity projectiles also create a temporary wound cavity in the bullet path as a result of radial tissue compression and cavity formation [9]. The presence of gas cavities allow a temporary wound cavity to be demonstrated radiologically in spatial relation to the bullet path. Our study showed that large-caliber bullets must be assumed to create larger temporary wound cavities than small-caliber bullets. This can be explained by the larger diameter and greater weight of the projectiles, which result in the transfer of a larger amount of energy to the target.

A loss of skin integrity in association with a gunshot injury almost always leads to wound contamination by either the projectile itself or by pathogens that are present in the air or on the skin [15]. Our results showed no direct correlation between caliber size and the depth of infiltration of exogenous particles. A greater diameter of the temporary wound cavity is associated with a larger volume of the temporary wound cavity, which increases the suction effect following the passage of the bullet. Exogenous particles, however, did not penetrate the entire bullet path or the entire temporary wound cavity. Irrespective of the caliber used, we found almost no barium titanate particles at a penetration depth of approximately 100 mm or more. The barium titanate particles that we used in our study have a size similar to that of bacteria and thus allow conclusions to be drawn on a possible contamination of the bullet path with pathogens. Accordingly, bacteria are unlikely to contaminate the entire bullet path. This confirms clinical studies conducted by Fackler et al., who also reported only mild bacterial contamination of gunshot injuries with exogenous pathogens [23]. According to that, a series of 32 low-velocity gunshot wounds were reported by Neupert and Boyd [24]. In our study we found the same results for the low-velocity bullets. Also in this case, there was an incorporation of particle in the gelatin. Comparing the result of our comparative experimental study, the damage resulted from projectile penetration which caused wounds characteristic of both high and low velocity penetration. We can say that the wound contamination by low-velocity in comparison high-velocity are comparable.

Gas cavities are nevertheless formed as a result of the compression of tissue in the temporary wound cavity and can be regarded as evidence of irreversible tissue compression. In summary, we must critically address the question of whether investigations in gelatin can be transferred to complex tissues in terminal ballistics.

### Clinical relevance

This study provides modern treatment strategies for gunshot injuries.

## Conclusion

Gelatin is only of limited value for evaluating the path of high-velocity projectiles and the contamination of wounds by exogenous particles. There is a direct relationship between the presence of gas cavities in the tissue along the bullet path and caliber size. These cavities, however, are only mildly contaminated by exogenous particles.

## Conflict of interest statement

The authors declare that they have no competing interests.

## Authors' contributions

CS, MR, MS, HK, MRU and NCG conceived of the study and participated in its design and coordination. CS and MR made substantial contributions to data acquisation and conception of manuscript and drafted and designed the manuscript. NCG and MRU were involved in revising the manuscript. All authors read and approved the final manuscript.

## Funding

The article processing charges are funded by the Deutsche Forschungsgemeinschaft (DFG), "Open Acess Publizieren".

## Pre-publication history

The pre-publication history for this paper can be accessed here:

http://www.biomedcentral.com/1471-2482/12/6/prepub
